# Complementary annealing mediated by exonuclease: a method for seamless cloning and conditioning site-directed mutagenesis

**DOI:** 10.1080/13102818.2014.988094

**Published:** 2014-12-10

**Authors:** Shuhui Sun, Hao Huang, Yingchuan Billy Qi, Mengsheng Qiu, Zhong-Min Dai

**Affiliations:** ^a^ Institute of Developmental and Regenerative Biology, College of Life and Environmental Sciences, Hangzhou Normal University, #16 Xuelin Street, Hangzhou, Zhejiang310036, China; ^b^ Department of Anatomical Sciences and Neurobiology, School of Medicine, University of Louisville, Louisville, Kentucky, KY40292, USA

**Keywords:** complementary annealing mediated by exonuclease, seamless cloning, ligation-independent cloning, site-directed mutagenesis

## Abstract

Traditional cut-paste DNA cloning is often limited by the availability of restriction enzyme sites. Here, we described the complementary annealing mediated by exonuclease (CAME), in which the insert or vector fragment is amplified to carry sequences complementary to the other, and both fragments are modified by exonuleases to create directional single-stranded overhangs. The two recessed DNA fragments are joined through complementary strand annealing. The CAME is highly efficient for cloning the DNA of at least 12 kb and single DNA fragment out of a complex DNA sample. Moreover, the application of CAME greatly improved the efficiency of site-directed mutagenesis.

## Introduction

Classic DNA cloning techniques rely on restriction enzyme digestion and DNA ligation.[1] However, the use of this cut and paste cloning is often limited by the availability of appropriate restriction enzymes for inserts and backbone vectors. In some cases, additional restriction site(s) are introduced into the final construct along with the insert, which alter the sequence of the vector and affect the function of the target protein or the expressivity of the construct.[2]

Methods designed for seamless cloning of inserts without the use of restriction sites have been reported, including enzyme-free cloning,[3] overlap extension polymerase chain reaction (PCR),[4] uracil excision-based cloning [5] and exonuclease-based cloning.[6–13] As the methods described above all require PCR amplification of the inserts, the cloning will be infeasible when the inserts fail to be amplified. *In vivo* recombineering is robust for cloning of inserts as large as 52 kb; however, the expression of the recombinase and the sophisticated selection schemes have to be employed [14] and often non-specific recombination may occur when the DNAs contain repeat sequences.

Here we describe an exonuclease-based cloning method termed complementary annealing mediated by exonuclease (CAME) for seamless cloning. Not only efficient for the cloning without involvements of restriction enzymes, but also the application of CAME greatly improved the efficiency of site-directed mutagenesis. More importantly, CAME method is competent to sub-clone a single DNA fragment out of the DNA mixture; thus, the method is expected to complement the conventional recombineering cloning.

## Materials and methods

### Scheme of CAME

**Figure 1.**
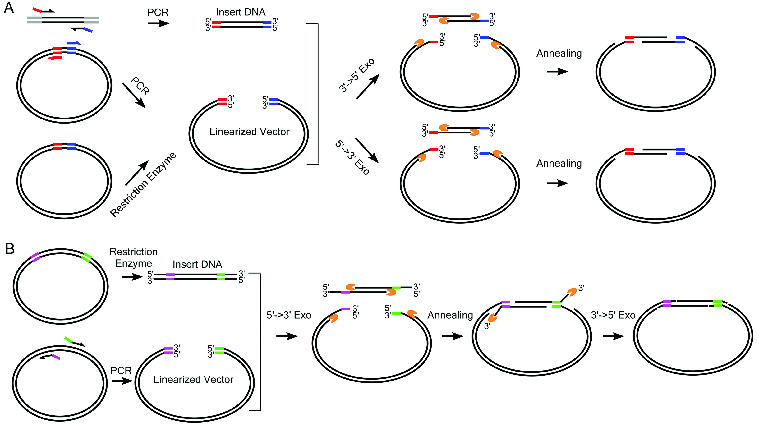
Complementary annealing mediated by exonuclease (CAME) for seamless cloning. (A) Insert DNA is amplified using PCR primers tailed by the sequences of more than 15 bp complementary to the backbone vector. Linearized vector can be generated by either restriction enzymatic digest or PCR. Enzymes either with 3′→5′ or 5′→3′ exonuclease activity are used to create single strand overhang. The two substrates are joined through annealing. (B) When PCR amplification of insert is difficult, insert can be generated by restriction enzyme digest, but the resulted fragment likely carries undesired sequences. Vector is then amplified using PCR primers containing sequences complementary to the insert. A sequential treatment with 5′→3′ exonuclease and 3′→5′ exonuclease ensures the formation of nicked circle. The gapped (A) or nicked (B) circle can be repaired after transformed into host cells.

As principle of the CAME procedure, the vector is either linearized by enzymatic digestion or PCR amplification, while the insert is amplified by PCR using chimeric primers, which introduces the amplified insert additional sequences complementary to the region flanking the vector cloning site (Figure 1(A)). Then, the vector and insert are mixed and incubated with T4 DNA polymerase, Pfu DNA polymerase or λ exonuclease. The treatment generates single-stranded complementary ends because of the enzymes’ 3′→5′ or 5′→3′ exonuclease activity (Figure 1(A)). Following the heat inactivation step, the vector and insert are further incubated at 50 °C for annealing. The annealed products are transformed into competent bacteria *Escherichia coli*. For certain cloning, PCR amplification of the insert may be difficult, e.g., due to its abnormal guanine-cytosine (GC) contents. To address this issue, we have adapted CAME for cloning the insert flanked by unwanted sequences (Figure 1(B)). Instead of amplifying the insert, we amplify the vector using chimeric primers containing complementary sequences to the ends of the insert, while the insert is prepared by restriction enzyme digestion. Then the mixtures of vector and insert are treated with λ exonuclease for producing overhangs and annealed. The unpaired protruding tails of the insert are trimmed by T4 DNA polymerase to form nicked circular DNA for transformation.

### Reagents

T4 DNA polymerase and T4 polynucleotide kinase (T4 PNK) were purchased from Fermentas. Pfu DNA polymerase was purchased from Sangon Biotech. Phusion DNA polymerase and λ exonuclease were purchased from New England Biolabs. Competent DH5α cells were prepared by the transformation and storage solution [15] or purchased from Beijing CoWin Bioscience.

### Measure of exonuclease activities

DNA fragments of 450 bp were used to test the exonuclease activities for the indicated enzymes (Supplemental Table S1). The 10-μL reactions were stopped by adding 2 μL of 6X loading buffer (30% (v/v) Glycerol, 100 mmol/L EDTA, 0.05% (w/v) bromophenol blue) and chilled on ice until agarose gel analysis.

### Preparation of vectors and inserts

Plasmid pBlueScript II KS (−) was used in this study. Vector was prepared by EcoRV digestion or PCR amplification of pBlueScript II KS (−). Primers used in this study are listed in Supplemental Table S2. Primer pair SGK2F & SGK2R were used to prepare inserts for comparison of CAME cloning using different exonulceases. Primer pair 12 kbF & 12 kbR was used to amplify 12 kb inserts from λ phage DNA. Primer pairs 0 bpP1 & 0 bpP2, 15 bpP1 & 15 bpP2, 20 bpP1 & 20 bpP2, and 25 bpP1 & 25 bpP2 were used to prepare vectors containing 0, 15, 20 and 25 bp sequences at the ends that are complementary to the 1.4 kb insert within a 2.2 kb AluI fragment of λ phage DNA. Primers pair UGAsecF & UGAsecR were used to introduce C258U mutation of firefly luciferase in the vector siCHECK-2 (Promega). Supplementary Figure S1 illustrates the PCR primer design rule for the CAME cloning with 3′→5′ exonucleases.

### CAME protocols

We routinely use 30–50 ng of the vector and 30–50 ng of insert for CAME cloning. CAME reactions were typically carried out in a total volume 10 μL with one unit of enzyme and its corresponding buffer (Supplemental Table S1). PCR products should be purified to eliminate dNTPs when Pfu or T4 DNA polymerase is used to recess the DNA ends. The program used for CAME were as follows: (1) for T4 DNA polymerase, recess the DNA using T4 DNA polymerase at room temperature for 3 min, heat inactivate the T4 DNA polymerase at 75 °C for 5 min and anneal the DNA at 50 °C for 20–30 min; (2) for λ exonuclease, recess the DNA using λ exonuclease at 37 °C for 5 min, heat inactivate the λ exonuclease at 75 °C for 5 min and anneal the DNA at 50 °C for 20–30 min; (3) for Pfu DNA polymerase, recess and anneal the DNA using Pfu DNA polymerase for 30 min at 50 °C; or (4) in cases of cloning of an insert whose ends were flanked by undesired sequences, recess the DNA using λ exonuclease at 37 °C for 30 min, heat inactivate the λ exonuclease at 75 °C for 5 min and anneal the DNA at 50 °C for 20–30 min, followed by using T4 DNA polymerase at 37 °C for 15 min to recess the 3′ overhangs and fill-in gaps.

## Results and discussion

### CAME for seamless cloning

**Figure 2.**
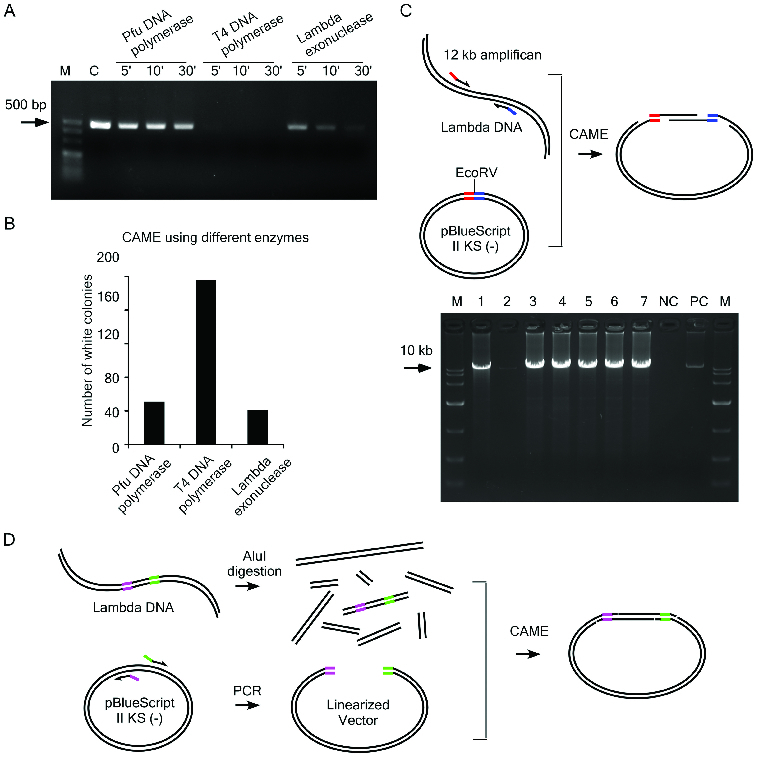
Test the efficiency of CAME cloning. (A) A 450 bp DNA fragment was incubated with indicated enzymes for various time periods. The weaker intensity of the bands resolved on agarose gel is correlated with the enzymatic reaction for longer period. C, the DNA without enzymatic treatment. (B) CAME cloning of a 1.1 kb insert using different enzymes. (C) Upper diagram illustrates the cloning of a 12 kb insert via CAME. Seven randomly picked white colonies (lane 1–7) were first screened by PCR amplifying the 12 kb insert and then verified by sequencing. NC, negative control using blue colony as template for PCR and PC, positive control using λ DNA as template for PCR. (D) Scheme for cloning of a 1.4 kb insert out of a DNA mixture.

As the annealing of the complementary single-stranded ends between vector and insert is predicted to be critical for the success of CAME, we devised an assay to estimate the exonuclease activities of T4 DNA polymerase, Pfu DNA polymerase and λ exonuclease by incubating a 450 bp DNA fragment with certain enzyme (see Supplemental Table S1 for detailed conditions). The assay was to find the conditions in which the exonuclease recession can adequately expose the complementary sites, while not to trim the whole DNA molecule too much. Our data indicated that T4 DNA polymerase exhibits the highest exonuclease activity and that it can digest almost the entire 450 bp DNA fragment in 5 min (Figure 2(A)). In comparison, λ exonuclease and Pfu DNA polymerase show intermediate and the lowest activity, respectively (Figure 2(A)). Based on these results as well as our empirical knowledge, we chose the CAME conditions as described in the ‘Materials and methods’ section. We tested the efficiency of the CAME by cloning a 1.1kb DNA fragment of SGK2 (see Supplemental Table S2 for primer sequences). The CAME using all three enzymes resulted in a large number of colonies (Figure 2(B)). The CAME using T4 DNA polymerase generates up to four times more white colonies than using the other two enzymes (Figure 2(B)). To test the CAME's efficiency on a large DNA fragment, we next cloned a 12-kb fragment amplified from λ phage DNA into pBluescript II KS (−) (Figure 2(C)). We used specifically T4 DNA polymerase in this procedure. From a transformed plate with 38 white colonies, 7 colonies were randomly picked and 6 of them were positive by PCR screening (Figure 2(C)) and later verified by sequencing. This result indicated that CAME can be used for cloning a large insert with high efficiency. The CAME cloning is much cheaper than the commercial available In-Fusion cloning (Clontech Laboratories), which involved an undisclosed enzyme for their reaction.

We next challenged the CAME cloning system to clone a single insert out of a mixture of DNA fragments (Figure 2(D)). Since there are 144 AluI sites in the λ phage DNA, digestion of λ phage DNA with AluI generates over 100 DNA fragments. A stretch of DNA sequence of 1.4 kb was selected as our target and the insert is flanked by about 400 bp of undesired sequences at both AluI-cut ends (Supplementary Figure S2). We found that the CAME treatment by a single exonuclease such as T4 DNA polymerase and λ exonuclease was ineffective for cloning the target fragment (Table 1). We then tested various combinations of the enzymes and found that the sequential treatment with λ exonuclease and T4 DNA polymerase generated the highest number of colonies with correct insert (Figure 1(B) and Table 1). To test the minimum length of complementary sequences required for the success in the cloning, primers tailed with various overlapping sequences with the target insert were used to amplify the vector (Supplemental Table S2). We found that complementary sequences longer than 20 bp are required for this cloning (Table 1). The success in cloning a single insert from a complex DNA samples suggests that CAME can be adapted for seamless cloning of a target from a large genomic clone, such as fosmids and bacterial artificial chromosomes (BAC) without PCR amplification of the insert.

**Table 1.** Cloning a single DNA fragment out of a complex sample.

Overlap (bp)	First enzyme(s)	Second enzyme	White colonies	Positive/screened colonies
25	−	−	6	1/6
25	λ exonuclease + T4 PNK	−	12	0/8
25	T4 DNA polymerase	−	2	1/2
25	λ exonuclease + T4 PNK	T4 DNA polymerase	32	5/16
20	λ exonuclease + T4 PNK	T4 DNA polymerase	29	2/16
15	λ exonuclease + T4 PNK	T4 DNA polymerase	21	0/16
0	λ exonuclease + T4 PNK	T4 DNA polymerase	31	0/8

### CAME greatly improves site-directed mutagenesis

**Figure 3.**
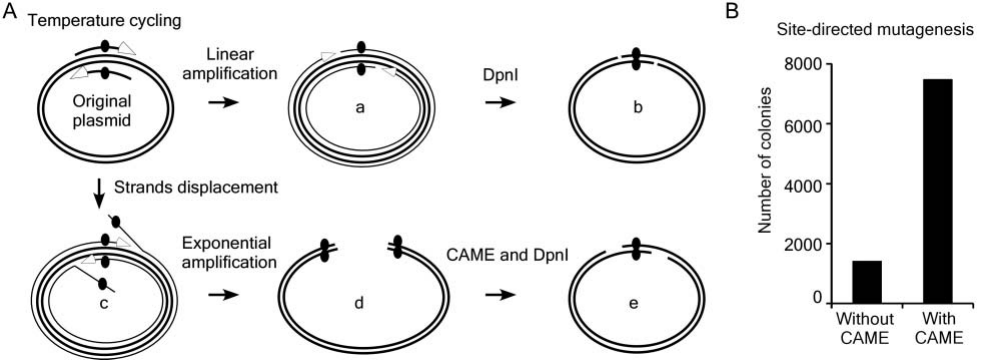
Improving site-directed mutagenesis using CAME. (A) Thermostable DNA polymerases possess weak strand displacement activity, which results in strand displacement (c) and exponential amplification of erroneous products (d) during site-directed mutagenesis. Such products will anneal and interfere with correct mutant DNA (b) to further reduce the mutagenesis efficiency. These erroneous products (d) can be converted into correct mutant (e) via CAME and DpnI treatment steps. (B) CAME improves site-direct mutagenesis by approximately fivefold in a point mutation of siCHECK-2.

Taking advantage of CAME's recessing and annealing properties, we applied the CAME in site-directed mutagenesis. Site-directed mutagenesis [16] is a linear amplification procedure that uses a PCR-like temperature cycling. For successful formation of relaxed circle with target mutation (type ‘a’ reaction in Figure 3(A)), the DNA polymerases used in site-directed mutagenesis must not possess strand displacement activity (‘c’ in Figure 3(A)). High-fidelity DNA polymerase, however, often possesses weak strand displacement activity during temperature cycling.[17] The type-‘c’ reaction is likely a common product during temperature cycling (Figure 3(A)). Type-‘c’ products, which can be exponentially amplified, may inhibit the formation of correct target mutants (‘d’ in Figure 3(A)) by annealing to them. We proposed that type-‘d’ products can be reverted to the correct mutant DNA (‘e’ in Figure 3(A)) if being treated with exonucleases such as T4 DNA polymerase. We tested this hypothesis by comparing efficiency of the site-directed mutagenesis with and without the treatment of T4 DNA polymerase. The TGC encodes Cys258 for firefly luciferase of siCHECK-2 (Promega) was mutated into TGA (stop codon or encodes Sec in case there is a cis-acting selenocysteine insertion sequence) using primers UGAsecF & UGAsecR (Supplemental Table S2) and Phusion DNA polymerase for site-direct mutagenesis. Both reactions were digested by DpnI, but one of them was treated with the CAME procedures (T4 DNA polymerase in this case) before transformation. In contrast to the 1410 colonies resulted from the control site-directed mutagenesis, 7474 colonies formed from the mutagenesis with a T4 DNA polymerase treatment (Figure 3(B)). Five colonies from each of the two cloning plates were randomly picked and verified to contain correct mutation by sequencing. We then CAME treat the DNA directly amplified from the siCHECK-2 containing *E. coli* cells with shortened primers UGAsecF2 & UGAsecR2 (Supplemental Table S2), and transformed them into competent cells without the DpnI treatment. Ten out of 10 randomly picked colonies were verified to contain correct mutation by sequencing (data not shown). The results demonstrated that CAME greatly enhances the efficiency of site-directed mutagenesis.

## Conclusions

In summary, our data showed that CAME provides a simple, effective and versatile procedure for seamless cloning of a DNA fragment into virtually any positions of a vector. We have also demonstrated that CAME treatment by T4 DNA polymerase can greatly improve the efficiency of the site-directed mutagenesis. Moreover, CAME succeeded in cloning a single target DNA fragment out of complex DNA samples, showing promise of a simple method for subcloning DNAs from a large molecule such as fosmid and BAC.

## Disclosure statement

No potential conflict of interest was reported by the authors.

## Supplemental data

Supplemental data for this article can be accessed http://dx.doi.org/10.1080/13102818.2014.988094
